# The Judge and the Doctor

**Published:** 1893-01

**Authors:** 


### Editorial, Etc.

THE JUDGE AND THE DOCTOR.—A most extraordinary decision, says the New York *Medical Record*, has recently been given by Judge Levy, of California, in the case of Dr. C. Ellenwood, who had a claim of $30,000 for medical services rendered to the family of a deceased millionaire. The executors of the estate and the three heirs all allowed the bill, but the Judge thought it too much and cut it down to $10,000! The Judge says in his opinion: "Although inexperienced in the charges of medical gentlemen for professional services, I am at a loss to recall a case where such an amount has been charged. I am of the opinion that the services rendered for one year's term were not of the value of $30,000, but a fair, reasonable and just compensation would be $10,000, for which amount this claim is allowed." Yet lawyers charge a much larger fee for services of much less intrinsic value. We hope that Judge Levy may be taken pleasantly and chronically ill, and then that Dr. Ellenwood may be called to attend him.

